# Genetic Polymorphisms of *ORAI1* and Chronic Kidney Disease in Taiwanese Population

**DOI:** 10.1155/2014/290863

**Published:** 2014-03-17

**Authors:** Daw-Yang Hwang, Shu-Chen Chien, Yu-Wen Hsu, Chih-Chin Kao, Shih-Ying Cheng, Hui-Chen Lu, Mai-Szu Wu, Jer-Ming Chang

**Affiliations:** ^1^Division of Nephrology, Department of Medicine, Kaohsiung Medical University Hospital/Kaohsiung Medical University, Kaohsiung 807, Taiwan; ^2^Department of Pharmacy, Taipei Medical University Hospital, Taipei 110, Taiwan; ^3^Department of Clinical Pharmacy, College of Medicine, Taipei Medical University, Taipei 110, Taiwan; ^4^Clinical Research Center, Taipei Medical University Hospital, Taipei 110, Taiwan; ^5^Department of Medical Genetics, College of Medicine, Kaohsiung Medical University, Kaohsiung 807, Taiwan; ^6^Division of Nephrology, Department of Internal Medicine, Taipei Medical University Hospital, Taipei 110, Taiwan; ^7^Graduate Institute of Clinical Medicine, College of Medicine, Taipei Medical University, Taipei 110, Taiwan; ^8^Department of Medical Genetics, College of Medicine, Taipei Medical University, Taipei 110, Taiwan; ^9^Department of Internal Medicine, School of Medicine, Taipei Medical University, Taipei 110, Taiwan; ^10^Department of Medicine, Kaohsiung Municipal Hsiao-Kang Hospital, Kaohsiung Medical University, Kaohsiung 807, Taiwan; ^11^Faculty of Renal Care, College of Medicine, Kaohsiung Medical University, Kaohsiung 807, Taiwan

## Abstract

Taiwan has very high incidence and prevalence of chronic kidney disease (CKD), which easily progresses to end-stage renal disease (ESRD). The association between inflammation and CKD has been explored in several studies. *ORAI1* functions as a pore-forming subunit of the store-operated calcium channels which are involved in the regulation of immune system. Hence, we conducted a case-control study to determine whether the genetic polymorphisms of *ORAI1* gene is a susceptibility factor to CKD and its clinical features in a Taiwanese population. Five hundred seventy-nine CKD patients from a hospital-based CKD care program were included in the study. Five tagging single nucleotide polymorphisms (tSNPs) of *ORAI1* were selected from the genotyping data of the Han Chinese population from the HapMap project. Among these polymorphisms, rs12313273 was found to be significantly associated with elevated serum calcium levels, which has been linked to increased risk of death in CKD patients. To have a better management of serum calcium, we suggest that *ORAI1* polymorphisms might be used as a potential biomarker for initiating non-calcium-based phosphate binder in CKD patients in the future.

## 1. Introduction

Chronic kidney disease (CKD) is an important global public health concern because of its high incidence, prevalence, morbidity, and mortality [1]. According to the US Renal Data System (USRDS) report, Taiwan has the highest incidence and prevalence of end-stage renal disease (ESRD) [2]. The prevalence of CKD in Taiwan was 9.8–11.9% and owing to the differences in the data sources, study subjects, and definition of CKD, the reasons behind this high incidence and prevalence are multifactorial [3].

CKD has been well known to be associated with low-grade inflammation, endothelial dysfunction, and platelet activation, even among those in the early stage of CKD [4]. Serum levels of the proinflammatory cytokines, such as IL-1, IL-6, CRP, and TNF-*α* were significantly high in CKD patients [5–8], and these inflammation markers may replace albumin, which is currently used as the predictive marker for mortality, to predict patient outcomes [9].

Calcium signaling controls diverse cellular functions such as enzyme metabolism, muscle contraction, immune response, and cell cycle regulation [10, 11]. In nonexcitable cells such as T cells and B cells, immunological reactions are regulated via Ca^2+^ entry mainly through store-operated calcium channels [12].* ORAI1* consists of four transmembrane domains and functions as a pore-forming subunit of the store-operated calcium channels [13]. Functional analysis of* ORAI1-* (also called* CRACM1-*) deficient mice revealed dysfunction of mast cells and attenuation of cytokine (TNF-*α* and IL-6) release [14].

Recent studies on the genetic susceptibility and the progression of CKD have yielded promising results [15–17]. The results of a genome-wide association study showed that several loci were associated with CKD and estimated glomerular filtration rate (eGFR) [16]. The evolution of ApoL1 variants as survival factors may have contributed to the high prevalence of renal disease among African Americans [17]. To the best of our knowledge, there is no previous research established regarding the association between genetic polymorphism of ORAl1 and the severity of CKD in Taiwanese population. Therefore, in this case-control study, we examined the association of the* ORAI1* genetic polymorphisms with CKD susceptibility, eGFR, and serum phosphorus and calcium levels.

## 2. Materials and Methods 

### 2.1. Study Subjects and Data Collection

Five hundred seventy-nine unrelated CKD patients (323 (55.8%) men; age range, 18–90 years old; mean age, 61 ± 14 years old) were included in the study at the time of their enrolment for the CKD Care Program at the Kaohsiung Medical University Hospital, Kaohsiung, Taiwan; written informed consent was obtained from all patients. All included patients were >18 years of age, and their detailed clinical history was recorded as part of the CKD Care Program. The study protocol conformed to the Declaration of Helsinki and was approved by the Institutional Review Board of the Kaohsiung Medical University Hospital. Serum creatinine levels were calculated using a modified kinetic Jaffe reaction. eGFR was estimated using the abbreviated equation developed in the Modification of Diet in Renal Disease Study [18], and the cases were categorized according to the staging system described in the Kidney/Dialysis Outcome Quality Initiative Clinical Practice Guidelines for CKD: Evaluation, Classification, and Stratification [19]. The patients were divided into two groups according to their eGFR: patients with eGFR above 45 mL/min/1.73 m^2^ were classified as having early-stage CKD [3, 20, 21], whereas those with lower eGFR were classified as having late-stage CKD. In Taiwan, the “nationwide CKD preventive project with multidisciplinary care program” implemented by Health Promotion Administration divided CKD patients into “early” and “pre-ESRD” stages, according to the eGFR ≥45 mL/min/1.73 m^2^ or <45 mL/min/1.73 m^2^ [22]. Different treatment strategy and management plans are applied in those two groups. In our study, we divided patients into two groups as above to investigate the differences of genetic polymorphism. Their clinical history and biochemical data were recorded.

### 2.2. DNA Extraction

Venous blood was collected from the patients during medical visit, stored at 4°C, and processed on the same day. The blood was centrifuged to separate serum and cells. DNA extraction from the blood cells involved an initial treatment with 0.5% SDS lysis buffer followed by treatment with protease K (1 mg/mL, for the digestion of nuclear protein) for 4 h at 60°C. Total DNA was harvested using the Gentra extraction kit and was precipitated using 70% alcohol.

### 2.3. SNP Selection

**Figure 1:**
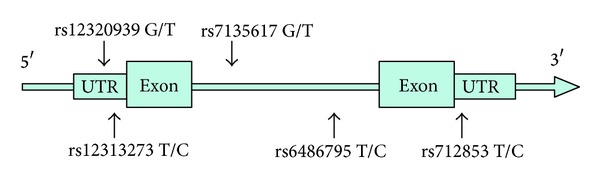
A graphical overview of the genotyped polymorphisms identified in relation to the exon/intron structure of the human* ORAI1 *gene.

From the HapMap database (http://www.hapmap.org, HapMap Data Rel 27 PhaseII+III, Freb09, on NCBI B36 assembly, dbSNP b126), five tagging single nucleotide polymorphisms (tSNPs) of* ORAI1* (rs12313273, rs6486795, rs7135617, rs12320939, and rs712853) with minor allele frequency (MAF) >10% and *r* ^2^ > 0.8 were selected from chromosomal region 120,545,838–120,561,329 of the Han Chinese population in Beijing (CHB). A graphical overview of the physical and chromosomal location of the five tSNPs is shown in Figure 1. Two* ORAI1* polymorphisms (rs12313273 and rs1232093) were located in the promoter region, two (rs6486795 and rs7135617) in the intron region, and one (rs712853) in the 3′-untranslated region (UTR).

### 2.4. Genotyping

Genotyping was performed using TaqMan PCR. In brief, TaqMan probes were first labeled with different fluorescent markers. PCR primers and TaqMan probes were designed to target the 5 tSNPs. Reactions were performed in 96-well microplates in the ABI 9700 Thermal Cycler (Applied Biosystems, Foster City, USA) and fluorescence was detected and analyzed using the System SDS software version 1.2.3.

### 2.5. Statistical Analysis

The genotype distribution of the five tSNPs was tested for Hardy-Weinberg equilibrium (HWE). The Chi-square test was used for comparing the genotype distribution or allele frequencies of the early-stage and late-stage CKD patients. One-way ANOVA was used to assess the difference in mean values of the eGFR and the serum levels of calcium and phosphate in the groups created based on genotyping results. All statistical analyses above were performed using the JMP 8.0 statistical software. Linear regression and logistic regression were used to adjust the influence of age in eGFR and CKD staging, which were performed using the SNPassoc 1.9-1 statistical software. A *P* value < 0.05 was considered significant.

## 3. Results

### 3.1. Association between* ORAI1* tSNPs and eGFR in the CKD Patients

Patient characteristics are shown in Table 1. We tested whether genetic polymorphisms in* ORAI1* are associated with eGFR in CKD patients. None of the tSNPs were found to be significantly associated with CKD susceptibility. We further adjusted our result by age which showed no significant associations (Table 2).

### 3.2. Association of* ORAI1* tSNPs in Early- and Late-Stage CKD Patients

Next, we evaluated whether the genotype and allele frequency of* ORAI1* were associated with the stage of CKD. After being adjusted by age using logistic regression, no association was observed between tSNPs and the stage of CKD (Table 3).

### 3.3. Association between the* ORAI1* Polymorphisms and Serum Calcium Levels in CKD Patients

Abnormalities in the levels of calcium, phosphorus, and intact parathyroid hormone (PTH) are evident early in CKD patients who are not on dialysis [19]. Since abnormalities in calcium and phosphate levels are associated with increased mortality and CKD progression in non-dialysis-dependent CKD patients [23, 24], we also investigated the associations between* ORAI1* genetic polymorphisms and serum calcium concentration. We found that rs12313273 was significantly associated with serum calcium levels in CKD patients (Table 4). We also observed that patients with the CC genotype of rs12313273 showed significantly higher calcium levels than those with other genotypes did. However, we found no correlation between the genetic polymorphisms and the serum phosphorus levels.

## 4. Discussion

We systematically investigated five* ORAI1* tSNPs (rs12313273, rs6486795, rs7135617, rs12320939, and rs712853) in CKD patients. None of the tSNPs of* ORAI1* were associated with the risk of CKD. However, rs12313273 was found to be significantly associated with increased serum calcium levels. Patients with CC genotype showed higher serum calcium levels than those with other genotypes. Impaired calcium and phosphate homeostasis have been reported in the early stages of CKD. We frequently used calcium-based or non-calcium-based phosphate binder to manage hyperphosphatemia, yet calcium-based binders often result in hypercalcemia [25]. Recent studies showed that CKD patients with high serum calcium levels (>2.75 mmol/L) have a higher risk of death than patients with low serum calcium levels do [26, 27]. Moreover, high calcium-phosphate product is associated with increased risk of vascular calcification and cardiovascular mortality [28, 29]. Our findings showed that patients with CC genotype of rs12313273 were associated with higher calcium levels. Therefore, we may take* ORAI1* polymorphism into account when prescribing calcium or non-calcium-based phosphate binder to CKD patients with hyperphosphatemia.

*ORAI1*-mediated calcium signaling plays critical roles in inflammatory diseases. Chang et al. identified several polymorphisms in* ORAI1* from Taiwanese and Japanese atopic dermatitis patients [30]. In addition, the CC genotype of rs12313273 in* ORAI1* was strongly associated with the risk and recurrence of calcium nephrolithiasis [31]. Furthermore, the* ORAI1* haplotypes (rs12313273 and rs7135617) are associated with the risk of HLA-B27-positive ankylosing spondylitis [32]. Consistent with the findings of previous studies, our results confirm the functional role of* ORAI1* polymorphism rs12313273 in modulating the serum calcium concentration.

The calcium-dependent pathway is involved in multiple physiological and cellular functions such as modulation of immune responses, activation of inflammation, and enzyme metabolism [33, 34]. Inflammation is an important mediator of CKD progression and is a contributing factor in malnutrition and increased risk of cardiovascular morbidity [35]. A vast body of evidence supports the important role of calcium in kidney disease. Mutations in transient receptor potential canonical 6 (TRPC6) channels and polycystin-2, a prototypical member of a subfamily of the TRPC channel superfamily, have been reported to cause familial focal segmental glomerulosclerosis and autosomal dominant polycystic kidney disease, respectively [36–41].

Recently, Lu et al. demonstrated a significant correlation between* TPRC1*,* ORAI1*,* STIM1*, and parathyroid cells [42]. PTH plays a key role in serum calcium regulation. PTH itself is also regulated by extracellular calcium through stimulating the calcium-sensing receptor (CaSR) expressed on the surface of parathyroid cells [43]. CaSR, a G-protein PLC-linked receptor, has been shown to be involved in the TRPC1-mediated transient calcium oscillation in human embryonic kidney cells [44]. Our results suggest that the genetic polymorphisms of* ORAI1* may alter* ORAI1* gene expression in store-operated calcium channels, which in turn may affect PTH secretion and thereby serum calcium levels.

This study has several limitations. First, we did not consider several factors that are known to influence calcium levels, such as concomitant drug usage and underlying disease. Second, the underlying comorbidities were not identified in this study, and a possible relationship between the different comorbidities and the tSNPs of* ORAI1* cannot be ruled out. Our results showed that the genotype of* ORAI1* was not associated with CKD susceptibility. However, owing to the moderate size of our cohort, our analyses may not have sufficient power for detecting minor genetic effects. Therefore, we cannot exclude rare causal genetic polymorphisms in* ORAI1*. Direct* ORAI1* sequencing using larger samples may be useful for identifying new SNPs in the* ORAI1* gene and for clarifying the association of* ORAI1* polymorphisms with CKD susceptibility. Further investigation on other variants of the genes of the SOC pathway and of the genes involved in calcium homeostasis are needed to fully understand CKD susceptibility and progression.

In conclusion, our results showed that the* ORAI1* polymorphism rs12313273 is associated with higher serum calcium levels in Taiwanese CKD patients. To have a better management of serum calcium,* ORAI1* polymorphism might be used as a potential biomarker for initiating non-calcium-based phosphate binder in CKD patients in the future.

## Figures and Tables

**Table 1:** Basal characteristics of patients with chronic kidney disease.

Characteristics	Patients with CKD
Number of subjects	579
Gender: male, number (%)	323 (55.8%)
Age (years)^a^	61.1 ± 13.7
Range (years)	18–90

^a^Means ± SD.

**Table 2:** Difference in the value of eGFR among CKD patients stratified by different *ORAI1* genotypes.

SNP	Genotype	Sample number (%)	eGFR^a^
rs12320939	TT	122 (21.2)	31.75 ± 24.28
	TG	295 (51.2)	32.26 ± 24.04
	GG	159 (27.6)	31.34 ± 24.28
*P* value			0.9229
Adjusted *P* value^b^			0.8764

	CC	50 (8.7)	34.64 ± 23.93
rs12313273	CT	245 (42.5)	31.08 ± 22.45
	TT	281 (48.8)	31.90 ± 24.70
*P* value			0.6229
Adjusted *P* value^b^			0.7209

	TT	98 (17.0)	29.06 ± 20.71
rs7135617	TG	285 (49.6)	32.71 ± 24.31
	GG	192 (33.4)	32.29 ± 25.44
*P* value			0.4138
Adjusted *P* value^b^			0.3691

	CC	71 (12.3)	33.49 ± 25.23
rs6486795	CT	271 (47.0)	31.79 ± 22.61
	TT	235 (40.7)	31.93 ± 24.90
*P* value			0.8619
Adjusted *P* value^b^			0.8608

	CC	56 (9.7)	34.36 ± 26.12
rs712853	CT	238 (41.4)	31.95 ± 24.39
	TT	281 (48.9)	31.53 ± 23.09
*P* value			0.7220
Adjusted *P* value^b^			0.7038

^a^Means ± SD. ^b^Adjusted age by linear regression.

**Table 3:** Genotyping and allele frequency of *ORAI1* gene in chronic kidney disease patients.

	Genotype	Late stage (%) (*n* = 453)	Early stage (%) (*n* = 126)	Allele	Late stage (%) (*n* = 453)	Early stage (%) (*n* = 126)	Genotype *P* value	Dominant *P* value	Recessive *P* value	Allelic *P* value
rs12320939	TT	94 (20.8)	28 (22.4)	T	424 (47.0)	115 (46.0)	0.6482	0.4482	0.7955	0.7363
	TG	236 (52.3)	59 (47.2)	G	478 (53.0)	135 (54.0)				
	GG	121 (26.8)	38 (30.4)							

rs12313273	CC	36 (8.0)	14 (11.3)	C	271 (30.0)	74 (29.8)	0.3780	0.5522	0.3148	0.9939
	CT	199 (44.0)	46 (37.1)	T	633 (70.0)	174 (70.2)				
	TT	217 (48.0)	64 (51.6)							

rs7135617	TT	80 (17.7)	18 (14.5)	T	378 (41.9)	103 (41.5)	0.4551	0.6285	0.3425	0.8567
	TG	218 (48.3)	67 (54.0)	G	524 (58.1)	145 (58.5)				
	GG	153 (33.9)	39 (31.5)							

rs6486795	CC	53 (11.8)	18 (14.2)	C	323 (35.8)	90 (35.7)	0.5856	0.6638	0.4536	0.9574
	CT	217 (48.1)	54 (42.9)	T	579 (64.2)	162 (64.3)				
	TT	181 (40.1)	54 (42.9)							

rs712853	CC	46 (10.2)	10 (8.0)	C	274 (30.4)	76 (30.4)	0.6072	0.6717	0.4667	0.9998
	CT	182 (40.4)	56 (44.8)	T	626 (69.6)	174 (69.6)				
	TT	222 (49.3)	59 (47.2)							

Late stage: eGFR <45, early stage: eGFR ≥45.

All *P* values had been adjusted by age using logistic regression.

**Table 4:** Difference in the value of Ca^2+^ and phosphorous among CKD patients stratified by different *ORAI1 *genotype.

SNP	Genotype	Sample number (%)	Calcium (mg/dL)^a^	*P* value	Phosphorous (mg/dL)^a^	*P* value
rs12320939	TT	122 (21.2)	9.32 ± 0.53	0.0528	4.26 ± 1.02	0.5243
	TG	295 (51.2)	9.10 ± 0.94		4.25 ± 1.02	
	GG	159 (27.6)	9.17 ± 0.80		4.37 ± 1.02	

rs12313273	CC	50 (8.7)	9.32 ± 0.61	0.0389*	4.33 ± 0.89	0.0831
	CT	245 (42.5)	9.23 ± 0.57		4.18 ± 1.01	
	TT	281 (48.8)	9.08 ± 1.03		4.38 ± 1.03	

rs7135617	TT	98 (17.0)	9.21 ± 0.87	0.1017	4.42 ± 1.05	0.3290
	TG	285 (49.6)	9.09 ± 0.81		4.24 ± 0.97	
	GG	192 (33.4)	9.25 ± 0.85		4.29 ± 1.07	

rs6486795	CC	71 (12.3)	9.33 ± 0.60	0.1586	4.36 ± 1.08	0.2622
	CT	271 (47.0)	9.17 ± 0.77		4.21 ± 1.03	
	TT	235 (40.7)	9.12 ± 0.96		4.35 ± 0.98	

rs712853	CC	56 (9.7)	8.99 ± 1.27	0.2356	4.47 ± 1.06	0.3133
	CT	238 (41.4)	9.16 ± 0.87		4.30 ± 1.12	
	TT	281 (48.9)	9.20 ± 0.68		4.24 ± 0.92	

*Significant (*P* < 0.05) values are in bold.^ a^Means ± SD.
